# Implementation context and stakeholder perspectives on routine immunization data among lower-level private for-profit providers in an urban setting: experiences from Kampala, Uganda

**DOI:** 10.1186/s12961-025-01351-7

**Published:** 2025-09-02

**Authors:** Eric Ssegujja, Paul Kiggundu, Sarah Zalwango Karen, Elizeus Rutebemberwa

**Affiliations:** 1 Department of Health Policy Planning and Management, School of Public Health, College of Health Sciences, Makerere University, Kampala, Uganda; 2 Directorate of Public Health and Environment, Kampala Capital City Authority, Kampala, Uganda

**Keywords:** Urban immunization, Data improvement, Private service providers, Kampala Capital City

## Abstract

**Background:**

Lower-level private for-profit health service providers form part of the pluralistic health systems delivering immunization services in urban areas of sub–Saharan Africa. However, their operational context is less documented since the conventional national Expanded Programme on Immunization (EPI) programmes tend to support delivery through public structures. Yet, private providers contribute greatly to immunization service coverage in urban settings. This paper explores the operational level context and stakeholders’ perspectives regarding immunization data among lower-level private for-profit service providers in the city of Kampala, Uganda. The objective of this baseline assessment was to document the current implementation context of immunization data among urban lower-level private for-profit immunization service providers to inform implementation research to improve immunization data in Kampala, Uganda.

**Methods:**

The study adopted an exploratory qualitative design where key informant interviews and in-depth interviews were conducted. Analysis was guided by the health systems building-block framework, which informed the design of the codebook with coding done in Atlas.ti, a qualitative data management software.

**Results:**

Overall, private for-profit immunization service providers reflected a context consisting of both barriers and opportunities underlying immunization data management practices. The barriers identified included: high staff turnover; data overload and manipulation tendencies; a transient population that access immunization services from different service providers without data linkage systems; computation of catchment populations, which affects utilization coverage data; financial barriers to the collection of community-level data; and inadequate facilitation leading to lean human resources at EPI departments managing immunization data from private providers. Nonetheless, opportunities to improve immunization data included the ability to widen data coverage through their services, enhanced public–private-partnership through data sharing arrangements, linkage of urban data among providers, improved recording of urban surveillance data, additional human resource to record data, widened scope for capturing adverse events data, improved community data linkages, and transitioning from paper-based to electronic data capture.

**Conclusions:**

Opportunities to improve urban immunization data management through private for-profit providers exist amidst numerous barriers. This calls for innovative strategies by the programme managers to design interventions with specific emphasis on addressing barriers inherent among urban lower-level private for-profit service providers if immunization data management among these entities is to be improved.

**Supplementary Information:**

The online version contains supplementary material available at 10.1186/s12961-025-01351-7.

## Background

### Overview of the National Routine Immunization data

Routine immunization is a strong pillar of the national public health services in Uganda and has contributed enormously to the reduction of morbidity and mortality from vaccine-preventable diseases [1, 2]. The national programme, the Uganda National Expanded Programme on Immunization (UNEPI), established under the Ministry of Health in 1983 oversees service delivery under a decentralized and tiered system [3]. Key players contribute to these efforts, including the public and the private sectors, with both private not-for-profits, under their respective medical bureaus, and private for-profits, as well as those run under non-governmental organizations (NGOs) [4]. Public facilities form the backbone of routine immunization whose programming heavily relies on routine data [5]. Under primary health care (PHC), the private sector is supported in the form of vaccines and data tools to deliver immunization services free of charge to service users with an expectation that they will collect and report utilization data into the District Health Information Software version 2 (DHIS2) for those with access, or report through the supervising public health facility for those lower-level service providers without access to the DHIS2 [6–8]. Under this arrangement, the Ministry of Health (MoH) through UNEPI supports the delivery of routine immunization through supporting semi-autonomous public facilities and the Directorate of Public Health and Environment under the city authority, as well as providing policy guidance, among others [5]. On their part, Kampala Capital City Authority (KCCA) supports routine immunization services by facilitating public health facilities, licensing and supervising private health service providers, and translating MoH policies, among others [9, 10]. 

### Private sector engagement to improve urban immunization coverage

The private sector is increasingly being seen as the most feasible alternative to supplement the lean urban public health service delivery systems to improve immunization coverage while addressing equity challenges. Broadly, the private sector is understood as the totality of privately owned institutions providing healthcare [11]. Their contribution is magnified by the high proportion of out-of-pocket health expenditure, which is a proxy indicator for the characteristics of private health sector suppliers [12]. The case for private sector involvement is also reflected in the heavy presence of private health suppliers, especially in urban areas [13]. Interlinkages in the form of referrals between public and private sector providers are another rationale for private sector involvement [14]. They have been seen as a key driver to realizing universal health coverage (UHC), with policymakers called upon to identify and ensure appropriate roles for private providers in health markets [14, 15]. Within the context of immunization service delivery, the private sector has supported national efforts through direct delivery of immunization services, offering sites for outreach, programme monitoring, adverse events reporting and monitoring, disease surveillance, data management improvement, and subnational level micro-planning, as well as working arrangements with the public sector in areas of cost sharing through provision of personnel and cold chain running costs [16, 17]. 

### Challenges with immunization data management among private service providers

Challenges with recording, processing, storage, and submission of immunization data from the private sector remain, and yet it is key in informing evidence-based planning for immunization services [18]. Whereas some stem from the business model through which private for-profit health services providers operate, others are context-specific, such as the urban nature in which health services are delivered, such as the transient population that access immunization services from different providers [6, 19], while others are thought to originate from existing collaborative arrangements with the public sector. Elsewhere, despite their complementation of the public sector in the delivery of health services such as immunization, the private sector has not been fully embraced to receive as much support from the central government as their public sector peers. Acceptance of, and confidence in, the private sector to deliver health services remains a contested area in driving the UHC agenda, with some viewing it as a betrayal of the public sector, which has long delivered these services [20]. Efforts to improve immunization coverage data have led to innovative interventions such as service user reminders and provider interventions [21]. 

Data recording and transmission denotes the ability of the system to collect accurate data and timely submission. Tremendous progress towards data management improvements has been registered in Uganda over the years. At its inception in 1983, Uganda’s National Expanded Programme on Immunization (UNEPI) data systems were typically paper-based, and by 1997, had been revised to include management indicators. By the onset of the Millennium Development Goals (MDGs) in 2000, further modifications were made, and since then, have been revised every 5 years. The year 2012 saw the introduction of the electronic system, which has greatly improved timeliness and completeness [3, 8]. Despite these improvements, immunization data is still faced with barriers to data management practices that continue to affect the planning and delivery of immunization services that typically occur during the recording, processing, and transmission stages. A recent study in Uganda revealed that tendencies of overreporting at lower-level health facilities were a common practice [3]. Similar cases of data duplication and overload have been reported elsewhere but at higher levels of the health service delivery structure [22]. Factors attributed to this include the low levels of data use, especially at lower levels, with weak mechanisms for quality assurance [23]. The situation is even worse among private providers, where many of the data systems remain underdeveloped, with low utilization for decision-making, due to poor quality, which further limits opportunities for data quality checks [24]. This is of special concern for Kampala where the private sector represents over 98% of health service provision in the city. The objective of this baseline assessment was to document the current context relating to the barriers and opportunities in the management of immunization data among urban lower-level private for-profit immunization service providers to inform implementation research to improve immunization data management practices in Kampala, Uganda. 

## Methods

### Study design

We employed a cross-sectional exploratory design utilizing qualitative data collection methods to document the current barriers and opportunities in recording immunization data among urban lower-level private for-profit immunization service providers within the city [25, 26].

### Study setting

The study was implemented in Kampala, Uganda’s capital city. It is the nation’s capital and the major business centre in the country. The city’s health service delivery system provided a case study where immunization services are predominantly delivered through the private sector. As reflected in Table 1, of the 1497 health facilities in the city, 94% are private for-profit providers, and the majority (94%) are at the HCII level (the lowest level of clinical service provision within the health system hierarchy). Overall, the city health system is typical of a pluralistic system with the presence of both public and private health service providers at different levels of health service provision [27]. Within the private sector, there are the private not-for-profits (PNFPs), which are faith-based; non-governmental organization (NGO)-based; and private for-profit (PFP)-based service providers [28]. The private for-profit sector is composed of high-level corporate hospitals and the lower-level health service providers (both licensed and unlicensed), often located in congested and informal settlements where public health facilities and high-level corporate hospitals are not present.

**Table 1:** Health facilities in Kampala by level of operation and ownership

Facility level	Ownership				Percentage by level
	Govt.	NGO	PFP	Total	
National referral hospital	4	0	0	4	0.27%
Hospital	3	6	14	23	1.54%
HCIV	1	2	8	11	0.73%
HCIII	6	17	30	53	3.54%
HCII	12	36	1358	1406	93.92%
Total	26	61	1410	1497	100%
Percentage by ownership	2%	4%	94%	100%	

Source: KCCA Directorate of Public Health and Environment Annual Report 2019/20

### Study population

Respondents were drawn from frontline health workers, health facility managers, immunization implementing partners, and the Capital City Authority public health managers. Mothers/caretakers of children were also included as immunization service users.

### Study sample and enrolment criteria

We purposively selected 25 respondents drawn from frontline health workers (*n* = 17), division EPI focal persons (*n* = 4), city health managers (*n* = 3), and immunization implementation partners (*n* = 1). Considerations were made for the hierarchical nature of the level of health service provision while accessing frontline health workers, with efforts made to access respondents from all of the city divisions. Potential respondents were approached through their workplaces by the research team members who introduced the study with the aims, objectives, and procedures for data collection clearly explained. For those who expressed willingness to participate, a favourable place and time were agreed upon when the interview was to be conducted. We did not collect data on response rates, but overall, all potential respondents that were approached to participate agreed.

### Data collection

Data was collected between April and June 2021 using an open-ended interview guide. The data collection tools were specifically developed for this study and have been attached as Supplementary Materials 2, 3, 4, and 5. Interviews were conducted by graduate-level research assistants using an interview guide developed by the research team following the objectives of the study and available literature. All interviews were conducted at the respondent’s places of work. On the day of the interview, a quiet and secure place was agreed upon and a digital audio recorder was used to record the interview. A notebook was used to record field notes during the interviews. Overall interviews lasted between 30 min and 1 h while focus groups lasted between 1 h and 1.5 h. All interviews were conducted in English, while the focus group discussions were conducted in Luganda, a dominant local language spoken within the city. These were transcribed verbatim into a Microsoft Office (MS) Word document. Focus group discussions were concurrently translated into English and transcribed following meaning-based translations.

### Data analysis

All transcripts were entered into Atlas.ti, a qualitative data management software, followed by the development of the codebook. The health systems building block framework was used as an organizing and analytical framework that guided the coding process, which was done by the first author and reviewed by the second author (P.K.). As coding progressed, emerging subthemes within each building block were coded inductively and added to the codebook as subcodes while maintaining the health system building blocks as parent codes. Within each block, the textual data relating to the barriers and opportunities were highlighted from the transcripts and attached to the corresponding building block. These were organized according to the levels of service provision, which included: community, health facility, sub-district, and the national level. After the coding exercise, query reports were produced from which a manual pile sorting exercise was done to group all texts that were similar into themes and subthemes. The results have been presented with typical quotations used to reflect respondent’s voices. Triangulation was done by comparing results from the different sources that were collected and levels of service provision. Finally, we followed the COREQ checklist to guide the presentation of results being reported here [29].

## Results

**Fig. 1:**
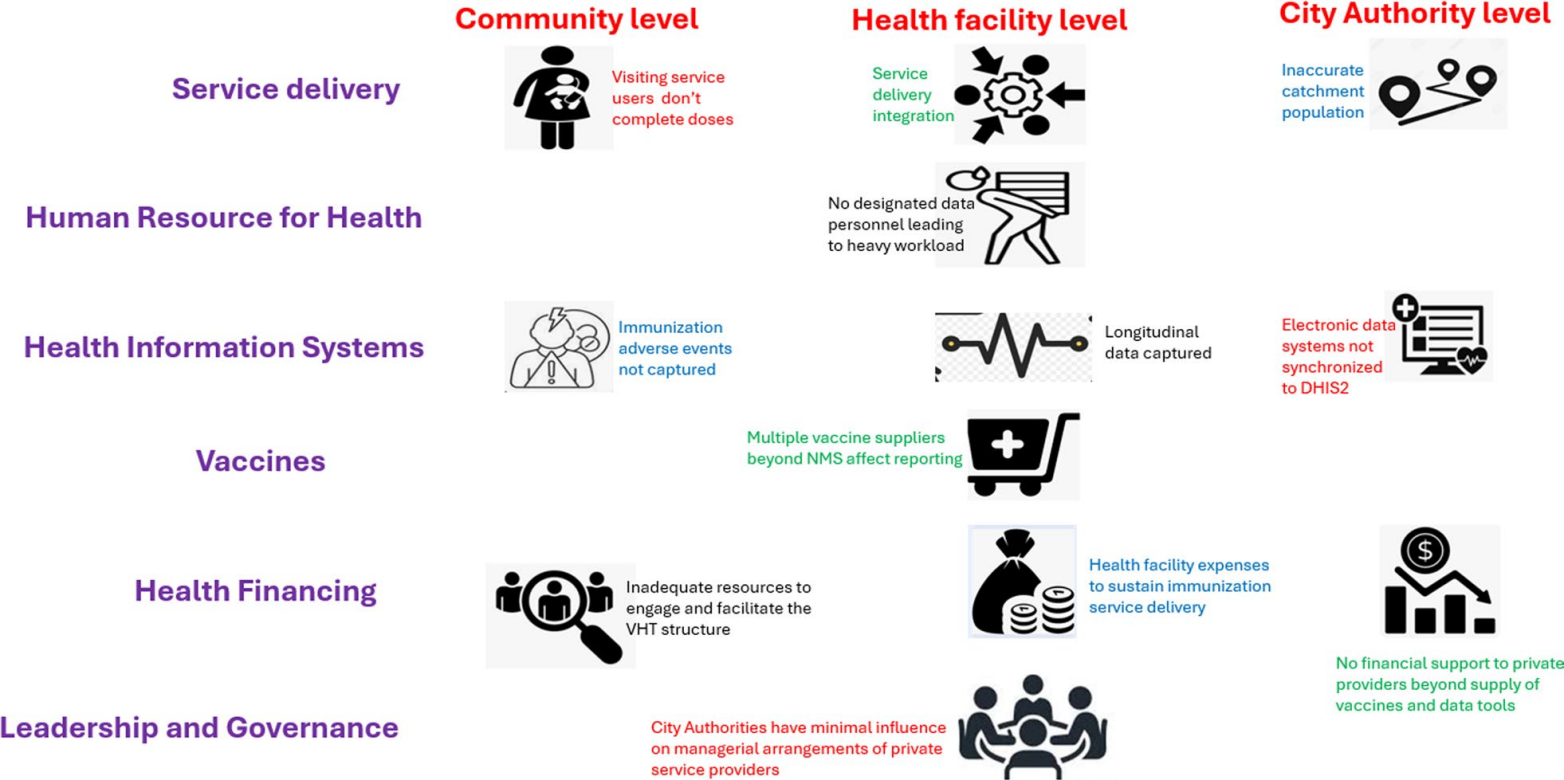
Immunization data processing levels according to the building blocks

Results from this study have been arranged according to the levels at which immunization data is processed, which include community level by the service users, the health facility level, and the city authority level up to the national level by the Ministry of Health. They are further presented by health system building blocks, which was the preferred guiding framework. These include: (1) health service delivery, (2) health workforce, (3) information systems, (4) essential medicines, (5) health financing, and (6) leadership and governance. The presentation follows a narrative format with themes and typical quotations. The illustration in figure one below reinforces the different data processing levels and typical implementation issues therein aligned according to the building blocks framework (Fig. 1).

### Health service delivery

#### Community level

Respondents reported the existence of a mismatch between the available urban immunization services and population characteristics and their preferences for immunization services. Many service users were reported to be visitors (children who are not part of the resident population of Kampala), while other urban dwellers were known to be transient, moving from one place to another, and yet individual private for-profit service providers lack cooperation arrangements with other service providers to facilitate tracking of lost-to-follow-up service users. After accessing the immunization services, particularly Polio 1 and DPT 1, they would continue with subsequent doses from their home districts, which affected immunization data capture due to the absence of a linking mechanism for subsequent dose uptake within the DHIS2 system. Such children would end up recorded as dropouts which reflected poor completion rates.

*We immunize over a 100% accessibility, only that by the time the children finish the three doses we may not have them all, some decide to go to the nearest immunization points, others just transfer. Kampala being an urban place, people keep on shifting and maybe sometimes that is the reason they do not complete [their vaccination schedules] with us, but according to DPT1 because we usually consider DPT when we have so many but by the time we reach DPT3 they have reduced. * (Health worker)

When many non-residents take up immunization services at the city’s lower-level private for-profit providers, it enhances the nation’s immunization coverage since rural districts are sometimes underserved, which is addressed by urban health systems bridging such a gap.

Similarly, linked services within the urban health system ecosystem improve service delivery access. Such information could be used to map out which entities were providing immunization, especially among the private providers. Even among the resident population of Kampala, many of the immunization service users who dwell in slum areas were reported to be highly mobile. Once they accessed initial immunization doses from a particular health facility, they would migrate and oftentimes accessed subsequent doses from health facilities within the communities they migrated to. This affected immunization data capture because of the lack of a linkage system to inform and update the data systems about their subsequent doses received elsewhere within Kampala.

*And then being a city centre facility in Kampala, we have always been faced with a problem of people changing location all the time where they stay, and so, we keep having very many people changing. Someone comes when they are getting their last dose, they are getting DPT3 and so you did not start with them, or you start with someone and then they say ‘I shifted I went to Nansana, I am no longer staying in old Kampala’ or something like that.* (Health worker)

#### Health facility level

Private providers had the potential for linked maternal and child health (MCH) services among private provider service users who may miss out on the same provided by public providers. Findings revealed that the organization of maternal and child health service delivery and data capture among private service providers led to barriers to the accurate capture and submission of immunization data. It emerged, for example, that tetanus toxoid vaccination administered during antenatal care (ANC) would oftentimes be captured although not be aggregated together with other immunization data recorded during the designated immunization days within the health facility while compiling vaccine utilization data.

*TT, the one they give to pregnant mothers, you find that the [health workers] will [administer it] on the antenatal side [and] not in the immunization side, so at the end when they are making the vaccine utilization and monitoring form you find that someone has sent in and he has forgotten the TT data, then you ask why, she will tell you ‘eh, that one is under the other [ANC side]’.* (EPI focal person)

A practice was observed among private service providers where they would administer vaccines that were not part of the MoH/UNEPI schedule. This was motivated by the demand from the service users. At private facilities where such vaccines were administered, the current tools used did not have provisions for recording such utilization data and hence loss of information from the national immunization data registries despite remaining at the particular private health facility. There remained the untapped potential to improve surveillance and monitoring of vaccine consumption patterns to establish the demand for such vaccines to use the information to inform national EPI programme planning.

*What I know about the international community. They know about immunization more than we the Africans, so they have their schedule they would prefer for us to follow. The Indian schedule when immunizing, so we sometimes do that and then there are some vaccines they prefer to get when they are not in the schedule, so we also try to get them… for those who will want to say ‘I want influenza vaccine’, ‘I want meningococcal’, which is not on the schedule, those are there… especially the Indian community.* (Health worker)

#### Subnational level

It emerged that the computation of the catchment population to determine coverage and inform planning for available immunization services remained a challenge. This affected data management practices where high default rates were reflected in the register. Kampala being a capital city and major business centre with a perceived urban advantage ended up serving the population outside of its catchment. The resultant effect on data was that of recording more than 100% coverage, and yet some children within the catchment still never accessed immunization services. It helps the urban centre not cover up defaulting, but demonstrates high demand in urban areas and user preferences for private providers as people move to these facilities to receive services.

*DHIS2 shows that central is performing well, but that is not a true picture because we also know that the expected population in central serves a bigger population than the expected resident population because the resident population for instance in the central division is much smaller than the people who access services.* (Implementing partner)

### Health workforce

#### Health facility level

Employment within the private sector provides for the absorption of excess human resources that could not be taken up by the public sector. Besides, tendencies of dual practice offers extra income for health workers to supplement their income. However, cases of heavy workloads were reported and partly attributed to the low staffing norms prevalent in many of the private health facilities. Sometimes, data entry tasks did not have specifically designated staff to handle. Consequently, it was either performed voluntarily or assigned to a health worker on top of their officially designated tasks.

*Yeah, one of us one time went and immunized and didn’t capture the information in the tally sheets so usually we get hardships in compiling the information and also balancing the stock of the vaccines that are given because most times you find you are alone and you have heaps of books and things may not move very well because you have to receive, register, immunize, and balance later so you may find that you may forget to tally sheets.* (Health worker)

Some health workers from the private immunization service providers were less motivated to record data, with less appreciation for its value in improving immunization services at the private health facility. This was partly attributed to a requirement for private facilities to have their data reported under the supervising public health facility from where they would pick their vaccines. It was reported to make it difficult for such private health facilities to analyze and appreciate their facility performance and motivate them towards aiming for more accurate data recording and timely submission. Private for-profit providers felt pressured to improve data management practices without appreciating the value of recording this immunization data, which translated into late submission and oftentimes inaccurate data.

*Sometimes, I think they are just reluctant, they don’t see the importance of reporting, so maybe every week they submit monthly reports but they never get feedback, they don’t like to see anyone following up on data so they don’t even see why they should report, so I think they don’t attach that much importance to reporting, maybe the way of making them realize that maybe reporting is important for planning, or a,b,c, they would, you know, also be motivated to report but some of them are not motivated to report.* (Implementing partner)

Whereas the many private for-profit providers were a source of employment to some health workers, their duration of stay was reported to be very short and some would move from one provider to another. The high staff turnover was a major barrier to the retention of skills necessary to improve immunization data recording and timely submission among the urban private service providers.

*… there is a challenge in private settings due to high staff turnover. So you find, like, I go and build capacity as pertaining our data tools, data reporting like that, and so within 2 months that person is off the job then is another one who doesn’t know anything completely but knows how to inject,… she is going to immunize and data may not be captured… but the truth is I think data reporting is a challenge more so for the private setting.* (Health worker)

### Health information systems

#### Community level

By working closely with village health team (VHT) members, private immunization providers widened their scope for capturing community-level immunization data, especially in urban areas where public sector service provision was lean. However, community-level data collection tools such as the VHT register, which collected immunization data, were never synchronized into the central data systems, thereby leading to loss of data.

*Having a tool of VHT register, all these tools like tally sheet books, do collect information that we need for immunization but, if we would have that so like where maybe we use systems because the government has DHIS2, where we always insert the data that we have collected from paperwork.* (Health worker)

#### Health facility level

Private for-profit immunization providers widen the scope for recording data on immunization adverse events. It emerged from this study that the system for capturing adverse events following immunization, although well-developed, was not communicated from the community level. While it was presumed to occur, very few parents managed to report this information. Even among those who made efforts to communicate such information, it was not well captured and communicated at the health facility level. A respondent thus observed:

*Another challenge we [are] faced with immunization services, you know there is an expected adverse effect after immunization, so very few parents report and even if they have reported they will move through the OPD department not through the immunization so they don’t communicate [and] so when you look at our data it looks like we have very few adverse events so those are the challenges.* (City data manager)

Private for-profit immunization service providers contributed to improvements in the utilization of data capture within the city. However, the data recording procedures adopted would sometimes lead to double reporting practices at some of the private for-profit health facilities. Findings reflected that whereas it was a requirement to report utilization data at the public facilities that supplied vaccines, for some private for-profit facilities entering their data directly into DHIS2 would also fulfil the mandate of reporting the same data through the supervising public facility, while at the same time reporting it directly through the DHIS2 as reflected in the quotation below:

*We were double reporting. We are reporting to DVS and then [HC], then they were like we are double reporting so we should stand on our own since we have the fridge we should report on our own. We are just picking the vaccines from [HF] but reporting to the DVS.* (Health worker)

Instances of data overload or manipulation during transmission were also reported as some of the undesirable practices engaged in at some of the private for-profit health service providers.

*… and you know private facilities some of them want to show that they are doing the work. They may bring up forged data and at the end of the day things are not tallying out, you see there is maybe over tallying maybe you are seeing the vaccines they took are not equivalent to what they are reporting, some of them bring requisition vouchers and some of them need calculations, and if you are putting wrong data the tool will show you that you don’t need vaccines yet you need vaccines.* (City health manager)

#### Subnational level

Being majority urban immunization service providers, many private for-profit health facilities were already using computer-based data capture that allowed for the gradual transition from paper-based immunization data systems to electronic-based data systems. However, in health facilities already with computer-based data capture, some tools were not developed in line with the data entry provisions compatible with the official HMIS form 105. This presented barriers to easy incorporation of data during transmission into the DHIS2. During the process, some data that was initially captured would get shed off in line with the DHIS2 compatibility, as reflected by a respondent in the quote below:

*Private facilities have already gone electronic, for us we are paper-based and our tools are designed on paper so you find that their electronic things and our papers do not match which affects the quality of the reports, we miss a lot of information like our registers capture a lot of details, yet if you notice their systems are just designed to track money, so they can get for you several patients immunized but they may fail to get for you dozes and you see that they are not compatible.* (City data manager)

Private for-profit immunization service providers with capacity (cold chain, access to computer, skilled human resource) entered their immunization data directly into the national DHIS2 systems. Some private providers were reported to delay submission of monthly data, which compromised the data management practices since the DHIS2 system would automatically lock out after the given submission deadline. Whereas the actual immunization event may have happened, the system would reflect no data assigned to that facility when data was not submitted on time.

*… because there are timelines to fill in the system, they know the system will be closed anytime soon so they have to find that you get reports first before these paperwork people because the other one of paperwork has to fill in around 29 pages of 105 and then remember they are few… We have a book where they write because we say by the 7th of next month all they should have reported all the reports should be at the division.* (Health worker)

#### National level

The longitudinal nature by which immunization data was captured at the health facility level was another barrier, as it was deemed user-unfriendly. A case was cited of the tedious work by which health workers had to search through the registers to identify cases where data were first recorded, especially for children returning for subsequent doses. It was especially problematic locating entries among high-volume facilities with more child registers.

*You see how the register was made, it is difficult because it does not give you an allowance to look at this child per visit, they want you to follow that child from when you enter the person you have entered them which is becoming a problem to us… the immunization book, you are supposed to trace a kid from behind so, it becomes hard to compile the report, you have to trace the information from pages behind.* (Health worker)

Further, the tools used were reported not to be harmonized, which made data recording a tedious process that posed barriers to the completion of entries by the frontline health workers, the consequences of which occasioned data loss between the different tools:

*We still have very, very big gaps in data, if you notice the primary tools for capturing EPI are very many and are bulk, we have a tally sheet, we have a register, you have a vaccine control book, you have the HMIS105, so the tools are many, they would expect if a child came, you fill in the child health card, then after filling in the card you record the child in the register then when you give the vaccine you tally then after you now summarize in the monthly report, so most times the procedure is tedious.* (City data manager)

### Access to essential vaccines

#### Health facility level

Private for-profit immunization services providers had the option of procuring vaccines from alternative sources other than the public supplier, National Medical Stores (NMS). This allowed for shorter turnaround periods in case of vaccine stockouts, which would address urban immunization service delivery interruptions. However, the lower-level private for-profit providers that procured vaccines from alternative sources were also faced with challenges of reporting utilization data through the HMIS tools and the DHIS2. Some private for-profit immunization services providers were reported to withhold vaccine utilization data for the simple reason that the particular vaccines in question were not procured from the KCCA and hence saw no need to account to an entity that did not supply such vaccines, which compromised the management practices of vaccine utilization data within the capital city.

### Health systems financing

#### Community level

Private for-profit immunization providers, working closely with the urban VHT structures, operationalize the urban VHT structure and monitor mothers closely for maternal and child health conditions. Nonetheless, the financial implications of following up on these mothers by the VHTs made it difficult to address missing data from the communities. Consequently, it affected the management practices of immunization data, especially from mothers of children who did not return to the same health facility to receive the subsequent doses.

*We have the registers, and the new registers now have a provision for putting mothers’ contacts. So when a mother fails to turn up, you call and find out why she didn’t come… we don’t use VHTs to help with the follow-up because they have to be paid and this is a private facility which makes it hard.* (Health worker)

#### Health facility level

The provision of immunization services by the private sector permitted more revenue for the facility owners when mothers of children return for other complementary health services offered at a cost. Current arrangements between MoH/KCCA and the private immunization service providers were reported to be that private providers were supplied with vaccines and data tools for free of charge in anticipation that they would also deliver immunization services for free to the users. However, in the process of delivery, many private for-profit immunization service providers incurred running costs which were sometimes transferred to the immunization service users. This affected data management practices, especially where mothers would default due to lack of money to facilitate their subsequent visits or among those who opted to seek for subsequent vaccination services from multiple suppliers, hence leading to data capture distortions.

*Most times you find private facilities have to invest in the immunization services, yet ideally immunization services should be accessed freely but because private providers have to make certain investments, buy equipment, pay health workers, they usually charge a small fee which may deter away some people, but which is necessary for the private facilities to keep running.* (Implementing partner)

#### Subnational level

The EPI department was reported to be somewhat inadequately facilitated in performing routine data quality checks and mentorships among lower-level private for-profit immunization service providers. They sometimes would end up just waiting for whatever was collected and submitted to the system, leaving minimal opportunity for responding to such errors introduced during the data recording stage.

### Leadership and governance

#### Subnational level

The directorate for public health and environment, which is responsible for monitoring urban immunization service provision within the city, has minimal influence on managerial arrangements within the private for-profit health facilities beyond facilitating them with vaccines, cold chain, and tools in anticipation that they would submit back utilization data. Managerial decisions affecting the delivery of immunization services among private providers would sometimes be taken when informed by resource availability. This affected data management practices in a way since some of the governance and management practices directly affected immunization data capture, and yet the city Expanded Programme on Immunization (EPI) department had less influence on decision-making to address such challenges. A case in point was the high staff turnover due to job insecurity and limited resource envelope among some of these private for-profit immunization service providers.

*These are private settings that are paying their workers and you can’t put a thick hand on them for example. The truth is that we say all static stations are supposed to immunize five times a week, like every day of the week if possible, but you find that these private facilities have UNEPI fridges but they are hesitant to offer immunization daily.* (EPI focal person)

## Discussion

Our results provide insights into the everyday barriers and opportunities for the improvement of immunization data management practices among lower-level private for-profit immunization service providers within Kampala city. Application of the health systems building blocks framework to understand these barriers and opportunities revealed that they transcend health management information systems. On one hand, the barriers are a proxy for the unfavourable contextual experiences at the different service delivery levels where data is recorded, processed, and transmitted. On the other hand, opportunities were reflective of the great contribution that the private sector makes towards the improvement of the data management practices of the national immunization data systems.

Overall, included incomplete data was due to high dropout rates owing to the transient nature of the immunization service users, the absence of a linkage/tracking system for cases that completed their subsequent doses from elsewhere, different data capture points within the same facility (which affected compilation), and data loss on vaccines outside the UNEPI schedule that were captured but not transmitted. Other barriers included difficulty in computing the catchment population leading to overreporting; high staff turnover, which affected skills for data recording/low motivation; and delayed submission. Instances of double reporting for some health facilities, which reported directly via DHIS2 and at the same time through their supervising health facilities where they would collect their vaccines, as well as tendencies of data overload and manipulation from some health facilities to justify vaccine quantities required. Financial barriers also affected data management practices in cases where community immunization data were not adequately collected/recorded, VHT registers were not synchronized with DHIS2, and when inadequate facilitation to the EPI department to facilitate supervision of data management practices among private for-profit providers, as well as health facility running costs, which were sometimes transferred to users, thus affecting completion rates.

Opportunities, on the other hand, were several, and these ranged from: enhanced public–private partnerships in immunization data collaborative aspects, linked/tracked immunization services and data, improved urban surveillance, provision of additional human resources for data capture, the widened scope for capturing immunization adverse events, and community data linkage, as well as the opportunity for transitioning from paper-based to electronic immunization data systems. This suggests that in addition to the benefits already accruing from engaging the private sector in the delivery of urban immunization services, a lot more could be achieved when the highlighted opportunities are fully embraced to respond to and improve the data management practices of urban immunization data. Efforts will need to be focused on the urban health system as a whole, as opposed to cherry-picking from the dysfunctional components that were not compatible with urban health management information systems. Through these results, we were able to learn that most of the opportunities shared by the respondents appeared to emerge from the implementation context within which the private sector immunization service providers delivered these services.

Specifically, we observed that barriers within immunization service delivery, such as the expected catchment population versus the actual immunization service users, were essential in facilitating the planning of management practices while handling the immunization data. Similar barriers have been reported elsewhere, which prompted the use of mapping to improve immunization coverage [30, 31]. The WHO, through the Global Routine Immunization Strategies and Practice (GRISP) programme, has laid out guidelines through which immunization service delivery can be improved [1]. Adopting such strategies in an urban context can go a long way in improving data recording from the lower-level private for-profit service providers.

Results revealed that whereas urban immunization services were planned for only the resident population, even visitors from neighbouring districts ended up utilizing these services. Consequently, the calculation of coverage would return with performance above 100%, and yet some urban dwellers never accessed these services. These findings have been consistent in Kampala [9], as has been reported in urban centres elsewhere [32]. It is important that an accurate catchment population is established to plan adequately for immunization service delivery and expected utilization performance within the city.

Aspects related to human resources were crucial in influencing the management practices of data recording and transmission. We observed that high staff turnover among the lower-level private providers hindered skill acquisition and retention, inadequate skills, heavy workload, and staff motivation, which all affected the immunization data management practices among private for-profit immunization service providers. For contextual reasons, we believe it affected the tracking processes of available immunization data records, consequently leading to compromised data management practices. Similar findings have been reported in Zimbabwe, where high staff turnover affected the delivery of immunization services [33]. The human resource challenges observed seemed to be peculiar to primary-level private for-profit health service providers. Given their implementation context, job security, and uncertainties surrounding their income seemed to drive these practices, leading to high staff turnover. It is therefore ideal that among the areas of support from the public sector, capacity building, specifically as it relates to governance and management of private health enterprises, would be useful. Current arrangements reveal that the public sector support to private providers is more concentrated around strengthening private provider capacities in technical aspects such as streamlining cold chain infrastructure and provision of vaccines and data collection tools. However, we observed that even when data collection tools were provided with mentoring around their use, immunization data management practices would still be affected by management and governance practices, which led to high staff turnover and less prioritization of immunization data, underscoring the importance of management and governance strengthening if data management practices are to be improved.

Leveraging the private sector presence has the potential to substantially improve the urban immunization service coverage and data management practices given their dominant presence in urban health ecosystems [11, 13, 15]. One aspect revealed from this study was that some bottlenecks to immunization data management practices, such as recording and transmission, stemmed from the way their data systems were organized. It emerged that the current tools used to collect immunization data were too many, and given the staffing norms at many of the private immunization service providers, led to late submission and sometimes low completion rates and poor-quality data. Still, the longitudinal nature of the registers made it challenging for health workers to track individual cases of children upon return for subsequent doses. The multiple submission points of the various tools was another issue raised, as well as the value of data in the business model of the private immunization service provision. Similar observations were reported elsewhere, where it emerged that the timeliness and completeness were partly due to the organization of immunization data systems [34]. Efforts to address immunization data management practices therefore go beyond facility-level operational factors to addressing long-standing barriers such as the organization of the data collection tools.

The national health services governance arrangement is anchored on the decentralized system, which extends to leadership and governance within the health sector. This arrangement in a way affected the planning and delivery of health services at the subnational level, including immunization services. Each local government administrative entity (including districts and urban authorities), including Kampala with its special administrative status, planned for delivery of health services in consideration of the resident population. However, this affected service delivery since many of the service users were not resident populations but rather visitors from other neighbouring districts. It affected immunization data in multiple ways. Once such service users received their initial vaccination doses, they would receive subsequent doses from elsewhere, either outside the city or at another immunization service provider within the city. There being no mechanisms to link data between the different service providers, it affected the data management practices, specifically the completion rates. It also affected the planning processes wherein, during the calculation of the catchment population, other service users residing outside Kampala would end up utilizing the immunization services, hence affecting the expected denominator from which effective coverage would be derived. In related ways, it affected immunization data while calculating the dropout rates. As we conclude, the results from this study have important public health implications for the improvement of immunization data management practices within urban settings. Sub-Saharan Africa is experiencing unprecedented levels of urbanization, with formally smaller townships developing into bigger commercial cities. According to current projections, more than 1.34 billion people are projected to be living in urban centres in Africa by 2050 (35). The number of megacities in Africa is projected to increase [36]. The urban health ecosystems have a predominantly private sector outlook [11, 15]. This means that improving immunization services within the urban context will involve prioritizing these private-sector players [16, 37]. The role of data in informing evidence-based planning for effective service delivery will be even more paramount. Interventions aimed at improving data systems within the private sector therefore, would benefit a lot from a clear understanding of the prevailing barriers and existing opportunities to inform the design of high-impact urban immunization data management practice interventions.

## Limitations

The findings from this study are at least subject to two limitations: the urban context within Kampala may vary significantly from other urban areas given the operational level factors affecting the delivery of immunization services by the private for-profit service providers. This may limit the generalizability of these results to other urban contexts. Secondly, social desirability may have been a factor in the provision of responses by the interviewees. With some participants being entrepreneurs of their private health facilities, they may have felt some discomfort in revealing some of the challenges they faced that had direct impacts on the services they delivered. Nonetheless, we had in place strategies to minimize all of these, and one of them was to ensure that interviewers explained well the aims and objectives of the study, and the other was a systematic triangulation of results across multiple data sources.

## Conclusions

Results from this study underscore the complex barriers and opportunities shaping immunization data management practices among Kampala’s lower-level private for-profit providers. Barriers, including incomplete data due to transient populations, high staff turnover, and inadequate catchment population estimates, reflect systemic health system challenges that transcend health management information systems. These issues, compounded by multiple data capture points, data loss, and financial constraints to deploy adequate staff, compromise timely and accurate reporting. Conversely, opportunities such as enhanced public–private partnerships, linked immunization tracking, and transitions to electronic systems highlight the private immunization service provider’s potential to complement efforts to strengthen national immunization data systems. The findings emphasize the need for holistic interventions addressing urban health system dynamics that include extending support to lower-level private for-profit service providers rather than isolated components focusing mainly on public health facilities and private not-for-profit service providers. Leveraging lower-level private sector presence, improving data tools, and building governance capacity can enhance data management practices. As urbanization accelerates in sub-Saharan Africa, prioritizing these strategies will be critical for evidence-based planning and equitable immunization coverage in urban settings such as Kampala.

## Supplementary Information

Supplementary material 1

Supplementary material 2

Supplementary material 3

Supplementary material 4

Supplementary material 5

## Data Availability

No datasets were generated or analyzed during the current study.

## Abbreviations

COREQ: Consolidated criteria for reporting qualitative results
DHIS: District Health Information System
EPI: Expanded Programme on Immunization
GAVI: Global Alliance for Vaccines and Immunization
GRISP: Global routine immunization strategies and practice
GVAP: Global Vaccine Action Plan
HC: Health centre
HMIS: Health management information systems
KCCA: Kampala Capital City Authority
MoH: Ministry of Health
NGO: Non-governmental organization
PNFP: Private not-for-profit
VHT: Village health teams
WHO: World Health Organization
