# Supplementary material for: Implementation context and stakeholder perspectives on routine immunization data among lower-level private for-profit providers in an urban setting: experiences from Kampala, Uganda
Source: Health Res Policy Syst. 2025 Sep 2;23:112. doi: 10.1186/s12961-025-01351-7 (PMC12406397; doi:10.1186/s12961-025-01351-7)
Supplement: Supplementary file 2 — Supplementary material 2 [file 12961_2025_1351_MOESM2_ESM.docx]

**Tool: Private immunisation services providers**

**Title of the proposed study:** Improving urban Immunization coverage through private sector involvement and e-health initiatives in Kampala, Uganda

Dear sir/madam

My name is ……………………………………………………………………a research team member from Makerere University School of Public Health in conjunction with Kampala Capital city Authority on a study to improve data systems for immunization coverage and equity. You are being asked to participate because you were identified as a potential respondent that is working closely in delivery of immunization services in Kampala.

1. Would you share with us your experiences with regards to provision of immunisation services to mothers and capture of data
   1. Who are the main users and what is the completion rates as well as capture of that data
   2. Any charges involved in accessing these services
2. How is data on immunisation captured and how is it used to improve immunisation coverage and completion rates?
3. What is your reporting arrangements with KCCA or MoH regarding immunisation data
4. As private sector, how do you think your immunisation services and data capture can be improved without compromising your costs of operation?
   1. How can this be made feasible for the provider of services to poor communities living in informal settlements of Kampala?
5. Are you currently using any electronic medical records at your facility
   1. Context and what are the operational issues around e-health tech you are using?
6. How do you ensure that caregivers complete the immunisation routines of their children and that data is captured and submitted
7. How can mobile phones be used to boost the completion rates of immunisation services and capture of data
8. What are the implementation challenges around capturing of immunisation data
9. What suggestions do you have with regards to use of e-health technology to improve performance monitoring of immunisation services and quality of immunisation data
10. How can e-technology be leveraged to improve immunisation data capture from the private sector
11. What proposals do you have with regards to transmission of this data into the central HMIS systems?
