# Supplementary material for: Implementation context and stakeholder perspectives on routine immunization data among lower-level private for-profit providers in an urban setting: experiences from Kampala, Uganda
Source: Health Res Policy Syst. 2025 Sep 2;23:112. doi: 10.1186/s12961-025-01351-7 (PMC12406397; doi:10.1186/s12961-025-01351-7)
Supplement: Supplementary file 3 — Supplementary material 3 [file 12961_2025_1351_MOESM3_ESM.docx]

**Tool: Implementing partner**

**Title of the proposed study:** Improving urban Immunization coverage through private sector involvement and e-health initiatives in Kampala, Uganda

Dear sir/madam

My name is ……………………………………………………………………a research team member from Makerere University School of Public Health in conjunction with Kampala Capital city Authority on a study to improve data systems for immunization coverage and equity. You are being asked to participate because you were identified as a potential respondent that is working closely in delivery of immunization services in Kampala.

1. What is your experience in working with KCCA to improve immunisation coverage within the city?
2. How is the private sector included in the immunisation services that you support in the city especially in reaching the most vulnerable such as the poor living in slums?
3. Are there any e-health strategies that you support to improve immunisation coverage?
4. How can these be leveraged to improve coverage and completion rates in the among the private sector immunisation providers?
5. Would you kindly share with us some of the implementation challenges that hinder immunisation data capture in the private sector?
   1. How can these be addressed using e-health technologies and electronic registers?
6. How can e-health be used to improve performance monitoring of immunisation services in Kampala?
   1. What are your suggestions with regards to using e-health to improve immunisation data capture into the central HMIS systems?
