# Supplementary material for: Implementation context and stakeholder perspectives on routine immunization data among lower-level private for-profit providers in an urban setting: experiences from Kampala, Uganda
Source: Health Res Policy Syst. 2025 Sep 2;23:112. doi: 10.1186/s12961-025-01351-7 (PMC12406397; doi:10.1186/s12961-025-01351-7)
Supplement: Supplementary file 4 — Supplementary material 4 [file 12961_2025_1351_MOESM4_ESM.docx]

**Tool: Caregivers**

**Title of the proposed study:** Improving urban Immunization coverage through private sector involvement and e-health initiatives in Kampala, Uganda

Dear sir/madam

My name is ……………………………………………………………………a research team member from Makerere University School of Public Health in conjunction with Kampala Capital city Authority on a study to improve data systems for immunization coverage and equity. You are being asked to participate because you were identified as a potential respondent that is working closely in delivery of immunization services in Kampala.

1. Share with us your experience as regards the immunisation services for your children in this area?
   1. Who are the different providers of these immunisation services?
   2. What is your experience with accessing immunisation services from the private service providers?
   3. What kind of information do you provide when accessing these services
2. What are your opinions regarding the current immunisation service delivery models?
   1. How can these be improved to better suite your interests for optimal uptake?
3. In your opinion why do you think caregivers don’t want to immunise their children or complete their children’s doses?
   1. What can be done to ensure that caregivers complete the doses of their children?
4. What do you see as some of the challenges with regards to accessing the available immunisation services?
5. How are you using the immunisation card?
6. In your opinion, do you think sending you sms reminders can improve your adherence to the immunisation routine?
