# Supplementary material for: Implementation context and stakeholder perspectives on routine immunization data among lower-level private for-profit providers in an urban setting: experiences from Kampala, Uganda
Source: Health Res Policy Syst. 2025 Sep 2;23:112. doi: 10.1186/s12961-025-01351-7 (PMC12406397; doi:10.1186/s12961-025-01351-7)
Supplement: Supplementary file 5 — Supplementary material 5 [file 12961_2025_1351_MOESM5_ESM.docx]

**Tool: Public facility Immunisation services providers**

**Title of the proposed study:** Improving urban Immunization coverage through private sector involvement and e-health initiatives in Kampala, Uganda

Dear sir/madam

My name is ……………………………………………………………………a research team member from Makerere University School of Public Health in conjunction with Kampala Capital city Authority on a study to improve data systems for immunization coverage and equity. You are being asked to participate because you were identified as a potential respondent that is working closely in delivery of immunization services in Kampala.

1. Would you comment on the status of immunisation services and capture of data in this facility?
2. Who are some of the partners you work with to improve immunisation coverage, completion rates and data capture?
3. What are some of the strategies that you have adopted to ensure you improve on your current reach of immunisation services and data capture?
   1. How can these be extended to cover the private sector that is providing immunisation services too?
4. At the facility level, how are you working with the private sector to ensure immunisation coverage, completion rates and data capture are improved in this area?
5. What are some of the e-health interventions that you are currently using at this facility to improve data
   1. How can these be leveraged to improve immunisation data capture from the private sector
6. What are some of the implementation challenges related to immunisation data capture in the private sector?
7. What are some of the e-health platforms that are used to improve data capture in Kampala?
   1. How can they be used to improve performance monitoring for immunisation services especially from the private sector?
   2. What suggestions would you give in relation to using these platforms from the private sector into the central HMIS registry?
